# Real and Imaginary Dangers of Moving Pictures

**Published:** 1917-11

**Authors:** 


### Real and Imaginary Dangers of Moving Pictures.

So far as we have been able to observe, the sanitary dangers are, on the whole, less than for theaters, schools and churches. Any place where large numbers of human beings are brought together, inevitably magnifies the danger of contact infection of all kinds but the better class of cinematograph theaters are cleaner, better equipped to minimize the accumulation of infectious matter and attended by a cleaner and more healthy audience than most other places of meeting for any purpose.

Eye strain, a factor of importance for ragged pictures, and those shown from unsteady instruments, does not seem to be excessive under proper conditions, unless perhaps for those whose attendance is excessive and, even then, it is questionable whether it is greater than for any comparable form of amusement. The glaring lights at some of our highest grade musical entertainments and the subdued lights in many churches involve more eye strain than most of the better grade picture shows.

Waste of time, unwholesome psychic influence and extravagance are chargeable to picture shows to just about the same degree as to other forms of indoor recreation, even to reading. A properly chosen picture show includes valuable information as to current events, good literature, visualized, good comedy, travel and nearly all other items that may be found in periodicals of the better type, presented in an easy way to receive and to remember. Low grade picture shows are equally comparable to low grade reading matter. In other words, the charges against the cinematograph in these respects are charges against the individual but the former are more open to criticism and to control by public opinion and legislation. Given an individual with much leisure, not of a type of mind conducing to self improvement and not physically able or inclined toward out-door recreation, the picture show is probably the least objectionable form of amusement available. Even deliberate choice could scarcely confine it to objectionable features nor prevent a good deal of educational benefit. A person with any serious interest in life or even a variety of social interests can scarcely become a ''movie fiend'' and would, if there were no such shows, waste time in some other way. As to extravagance, even a fiend can scarcely spend more in a week than on a single evening of recreation of another kind.

Sexually objectionable shows, either in the sense of anatomic displays or of immorality of plot seem to be on the whole less frequent in films than on the stage. Perhaps, however, the mere fact that one is viewing a picture discounts the first element. The vilest plots, of either film or humanly acted dramas, are in problem plays exploited as educational and occasionally endorsed by uplift societies. We do not remember even to have seen a picture play that was as bad in the first regard as actual plays by living actors and, unless especially advertised as of moral value, very few picture plays are objectionable on the latter score.

We are somewhat sceptic as to the influence of picture shows in inciting to crime. As a rule, even when burglaries, combats, etc., are shown, wrong doing is punished or the criminal reforms. And it must not be forgotten that the ''lower'' the show and its audience, the greater is the tendency to emphasize trite moral conceptions and ethical ideals. It is hardly imaginable that a professional criminal could obtain pointers from a picture show. The amateur, would-be criminal may imagine that he is obtaining useful information for a career of crime but, if so, his detection is all the more certain. Young children may be unduly excited by almost any form of evening recreation, may start off to kill Indians or lead a buccaneer's life just as they did long before the cinematograph was invented but the normal boy and girl, of an age to have any conception of the real instruments and methods of crime, is now sophisticated to the point of taking fiction at its proper value. So far as we have observed, accounts of incitement to juvenile crime by picture shows have been much exaggerated and apply only to the mentally defiicient who ought to be under custodial control of some sort. However, these remarks are not to be construed as a defense of the kind of pictures discussed.

The real danger of the picture show, as of the stage and of fiction in general, is the conveyance of wrong information, all the more liable to be accepted at its face value because of the insidious, taken-for-granted way in which it is presented. For example, the third degree, a favorite topic, should be regarded from two extreme view points. If there is at present any basis of truth for such depictions, the matter ought to be investigated and the evil stopped. If the implication is false, such depictions should be penalized.

Physicians certainly cannot claim that our profession is neglected by the movies but one is inclined to speculate whether the conceptions that we gather as to other professions and industries are as far from the truth as the majority of implications as to our own work. Some of the medical teachings of the movies to which we particularly object are the following: That physicians commonly fall in love with their patients or nurses; that nurses "save the day" in spectacular ways in the conduct of patients, especially by interfering with the egregious blunders of physicians; that the hardships of a medical career are removed by one signal success—a mistake even more frequent in printed than in filmed fiction; that physicians, as political agents or private malefactors, use drugs, bacterial cultures or mere inforced neglect to kill human beings; that they are oppressive in their charges or, on the other hand, that indiscriminate free service is desirable; that it is a noble act, justified by superior skill and humanity to steal a patient; that under any ordinary economic conditions, physicians can maintain the mansions in which they are placed by film play wrights or that they can attend to practice at all in the irregular and brief intervals between yachting trips, prolonged vacations for other purposes, or engrossing political, scientific and social occupations. It is, to say the least, aggravating to those of us who gain a very moderate degree of diagnostic skill and therapeutic power by many years of hard labor, to read or view a film, the achievements of medical supermen. We envy the fiction doctor who pulls his patient through a crisis by some sort of mental exultation. We recall experience in many diseases and wonder how far even the conservative medical conception of a crisis is justifiable. We compare our own mistakes and confessed ignorance as to prognosis with the accurate predictions as to the duration of a sleep, of the fiction doctor. We wonder why we have not seen cases of "brain fever" like those of fiction. We note resuscitations from drowning and gas poisoning under conditions in which we would begin a necropsy. We are amazed at glib represensations of dual personality, metemsychosis, restoration to health—and usually marriage ability—of cases of epilepsy, melancholia and other grave neurotic affections and wonder if such representations may not have some effect on evil heredity. On the other hand, we are grateful at the relative frequency with which we enjoy a night's sleep without professional interruption, and the infrequency with which we make calls in blizzards or through swollen streams with the bridges down. We poor real physicians grow rusty very rapidly without continued experience. Not so the fiction doctor. The governor of a state, who early left medicine for a political career, is quite competent to attend to any emergency that may arise in a medical way. The doctor who has become a country gentlemen dispenses medical charity not only with absolute faith in his competence but without a qualm at encroaching on the practice of his neighbors. In real life, physicians rarely treat their own families, recognizing the disturbing influence of personal affection on even a high grade of skill in the condition involved. The real physician frequently violates this common sense rule. When a physician not in active practice is depicted in the foreground of a miscellaneous lot of electric and chemic junk, raising the dead to life, no protest is necessary as the matter is obviously in the realm of the supernatural and impossible. But, the same principle carried out to a minor degree, within the realm of the possible, does great harm, by arousing false hopes and establishing unattainable standards by which our profession will be judged.

It is particularly exasperating, at the close of a film that insidiously teaches falsehoods that may have a serious influence on popular conceptions to have flashed on the screen: "Passed by the Board of Censors." One wonders where the Board of Censors does draw the line.

